# IMPLEMENTATION OF BRAINCHECK FOR COGNITIVE ASSESSMENT AND CARE PLANNING IN THE CLINICAL SETTING

**DOI:** 10.1093/geroni/igad104.2946

**Published:** 2023-12-21

**Authors:** Bin Huang, Patrick Zavala, Christine Mann, Sarah Harlock, Kori Dekle, Amy Knighton, Bela Ajtai, John Hemphill

**Affiliations:** BrainCheck, Austin, Texas, United States; BrainCheck, Austin, Texas, United States; DENT Neurologic Institute, Amherst, New York, United States; DENT Neurologic Institute, Amherst, New York, United States; Savannah Neurology Specialists, Savannah, Georgia, United States; Savannah Neurology Specialists, Savannah, Georgia, United States; DENT Neurologic Institute, Amherst, New York, United States; Savannah Neurology Specialists, Savannah, Georgia, United States

## Abstract

With the growth of the older adult population (age ≥ 65 years), age-associated neurocognitive disorders, such as Alzheimer’s disease and related dementias (ADRD), are becoming more common in America and worldwide. The increasing number of patients has surpassed the capacity of healthcare systems. Thus, there is a need for time-efficient, cost-effective solutions for better allocation of limited clinical resources that can benefit clinicians and patients. Here, we conducted a quality improvement study in collaboration with Savannah Neurology Specialists and Dent Neurologic Institute. We evaluated users’ perspectives on the usability and feasibility of implementing BrainCheck, a computerized tool for cognitive assessment and care planning. One neurologist and their team from each institute received 45 minutes of training on BrainCheck and used it on an iPad from mid-November to the end of December 2022. In total, 49 patients have completed BrainCheck Cognitive Assessment, and 7 of them also completed BrainCheck Cognitive Care Planning during the study period. On average, it took 13-18 minutes to administer BrainCheck Cognitive Assessment, interpret the result, and document it in EHR. The majority of patients (96%) reported satisfaction with the testing experience and found the instructions easy to understand. Both providers reported high usability of BrainCheck (6.2/7.0) as measured by the Post-Study System Usability Questionnaire. These results demonstrate the great potential of BrainCheck for clinical use, as it can support providers in assessing the cognitive function of their patients in a timely and efficient manner.

